# Near-Infrared Spectroscopy Combined with Chemometrics for Liquor Product Quality Assessment: A Review

**DOI:** 10.3390/foods14172992

**Published:** 2025-08-27

**Authors:** Wenliang Qi, Qingqing Jiang, Tianyu Ma, Yazhi Tan, Ruili Yan, Erihemu Erihemu

**Affiliations:** College of Food Science, Shanxi Normal University, Taiyuan 030031, China; wlqi@sxnu.edu.cn (W.Q.); jqq19806235186@163.com (Q.J.); 13015237534@163.com (T.M.); 13834902117@163.com (Y.T.); yrl.com@139.com (R.Y.)

**Keywords:** near-infrared spectroscopy, chemometrics, liquor product quality, quantitative analytical modeling, artificial intelligence algorithms

## Abstract

China’s liquor industry continues to steadily expand and develop. The industry is currently transforming, shifting its focus from scale to quality and efficiency. This transformation is significantly increasing the demand for quality and safety testing. Currently, the testing system relies mainly on manual operation or traditional mechanical equipment. Technical bottlenecks include low testing efficiency, a significant imbalance in the cost–benefit ratio, and difficulty meeting the modern industry’s dual technical index requirements of testing accuracy and systematicity. In this context, the innovative research and development of new detection technology is key to promoting technological upgrades in the liquor industry. Near-infrared (NIR) spectroscopy is a core, competitive analytical method for non-destructive wine quality testing due to its technical advantages, such as non-destructive analysis, real-time online detection, and the absence of sample pretreatment requirements. This study systematically elaborates on the optical principle and detection mechanism of NIR spectroscopy and explores the application paradigm of chemometrics in spectral data analysis. This study covers the quantitative analysis of alcoholic strength, the determination of main ingredient content (sugar, acidity, esters, etc.), the construction of trace flavor substance fingerprints, the authentication and origin tracing of alcoholic products, and the monitoring of wine aging quality dynamics, among other key technology areas. Additionally, we review the fusion and innovation trends of artificial intelligence and big data technology, the R&D progress of miniaturized testing equipment, and the technical bottlenecks of spectral modeling and algorithm optimization. We also make scientific predictions about the evolution path of this technology and its industrial application prospects.

## 1. Introduction

Near-infrared (NIR) spectroscopy is based on the absorption properties of substances in the near-infrared region, and the data are collected with a spectrometer to analyze the chemical and physical properties of samples. This technique has been widely used in the fields of food [1,2], agriculture [3,4] and medicine [5,6] due to its advantages of non-destructiveness, rapidity, and accuracy.

In the field of alcohol quality assessment, near-infrared spectroscopy technology allows for the rapid determination of key indexes such as alcohol content, total sugar, total acid, and so on by resolving the differences in spectral characteristics of different alcohols [7,8,9,10]. The main advantage of this technique in the field of alcohol detection is that it can rapidly and accurately obtain the spectral information of alcohol samples, thus allowing for the rapid assessment of the quality of alcohol, and the method is non-destructive to the samples, which makes it particularly suitable for the detection of large-scale samples. However, the limitation of NIR spectroscopy is that the results are susceptible to a variety of factors such as instrumental accuracy, operating conditions, and sample state, which may lead to bias in the analytical results. In addition, in order to improve the accuracy and reliability of the analysis, the technique must be combined with chemometric methods for data processing and modeling [11,12].

**Figure 1:**
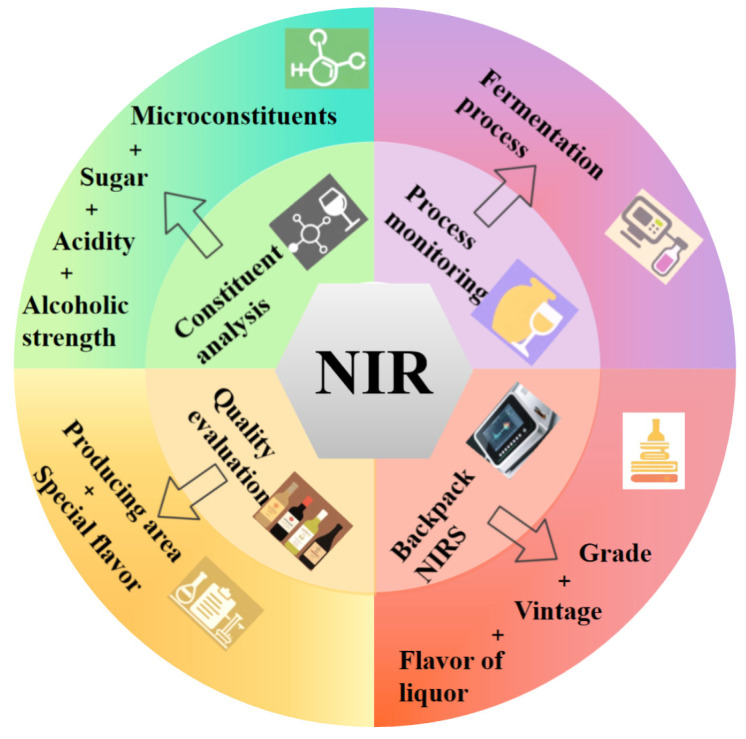
Applications of NIRS in liquor quality assessment.

Specific analytical models and assessment criteria must be constructed for the complex spectral properties of different alcohols such as white wine, yellow wine and red wine [13,14,15,16,17]. The accuracy of wine quality assessment depends not only on the accuracy of NIR spectroscopic techniques, but also on the in-depth understanding of the characteristics of various types of wine. For example, white wine is famous for its unique brewing process and rich flavor components, and its spectral response characteristics are complex and variable [18,19]; the brewing of yellow wine, a traditional Chinese alcoholic beverage, is affected by a variety of factors, such as the type of raw materials, fermentation conditions, and aging time, which may affect the spectral characteristics of the rice wine [20]; and wine exhibits diversified spectral characteristics due to its different places of origin, grape varieties, and brewing methods [21,22]. The spectral characteristics of beer are closely related to its raw materials, fermentation process and subsequent processing [23], while the spectral responses of spirits such as whisky and vodka are more reflective of their raw material characteristics and distillation process [24]. Therefore, the construction of specific analytical models and evaluation criteria for different liquors is the key to achieving the wide application of NIR spectroscopy in liquor quality assessment. The application of NIR spectroscopy in the quality assessment of wine is shown in Figure 1.

## 2. The Role of Chemometric Methods in the Processing of Near-Infrared Spectroscopic Data

### 2.1. Data Preprocessing Techniques and Their Impact on Model Performance

In the domain of near-infrared spectral data analysis, preprocessing the data, which encompasses spectral smoothing, filtering, denoising, baseline correction, and normalization, exerts a key influence on the precision and stability of the model’s predictive outcomes. Wang et al. [25] achieved a classification accuracy exceeding 99% for liquor brands through the integration of Principal Component Analysis (PCA), Linear Discriminant Analysis (LDA), Support Vector Machines (SVM), and Back-Propagation Neural Networks (BPNN). Nevertheless, this approach incurs significant costs when applied to multispectral data fusion.

Zhang et al. [26] carried out a qualitative analysis based on the response properties of NIR to hydrogen-containing groups combined with machine learning techniques. Finally, a hierarchical multi-class support vector machine method was used to establish a white wine quality recognition model based on principal component analysis. The model’s prediction accuracy reached 96.87%, and this fast non-destructive testing method successfully solves the problem of relying on subjective sensory evaluations for the analysis of white wine. Wang et al. [27] effectively solved the problem of the one-dimensional aliasing of spectral signals by applying multiscale computation, Savitzky-Golay (S-G) filtering, and the finite difference method for preprocessing in combination with two-dimensional simultaneous correlation spectroscopy techniques and a convolutional neural network. This significantly improved the accuracy of wine origin tracing. Pu et al. [28] developed a one-dimensional convolutional neural network (1D-CNN) for near-infrared spectral classification without spectral preprocessing, with an accuracy as high as 93.75%, which exceeds those of other methods such as those using back-propagation neural networks, support vector machines and extreme learning machines (ELMs). The preprocessing technique is crucial for improving signal quality, reducing errors, eliminating baseline drift, and enhancing prediction accuracy and generalization, and is a key step in wine quality assessment.

### 2.2. Qualitative Discriminant Model Construction and Validation Methods

Qualitative discriminant models are crucial for accurately identifying the authenticity, geographical origin, and specific type of wine, serving as vital tools in quality control and fraud prevention within the industry. Their application algorithms encompass decision trees, random forests, support vector machines, and other machine learning techniques that analyze complex datasets derived from chemical compositions and sensory attributes to deliver reliable and efficient classification outcomes. Zhang et al. [29] combined the Long Short-Term Memory (LSTM) network with multi-source data (gas chromatography, electronic nose, near-infrared spectroscopy) and successfully achieved yellow wine vintage classification (with an accuracy of 100%), as well as significantly improving the prediction accuracy of physicochemical parameters. Aiello et al. [30] compared three machine learning models, namely, random forest (RF), Linear Discriminant Analysis and K-Nearest Neighbors (KNN), in the assessment of wine quality (with an accuracy of >0.65) and origin traceability (accuracy greater than 0.99). Koljančić et al. [31] used NIR spectroscopy in combination with chemometrics analysis techniques to successfully achieve the accurate distinction of wine varieties.

### 2.3. Quantitative Analysis Model Construction and Optimization Strategy

Quantitative models, encompassing partial least squares (PLS), SVM, and artificial neural networks (ANNs), have been extensively utilized to establish correlations between indicators of wine quality and spectral data. Harris et al. [32] integrated near-infrared spectroscopy with electronic nose technology and employed machine learning models to classify wine vintages with an accuracy of up to 98.3%. Silva et al. [33] implemented an artificial neural network to classify wines with an accuracy of 95.2%, surpassing the 88.9% achieved by discriminant analysis. Kolobaric et al. [34] applied the PLS method to detect methanol adulteration in whisky. They found that the method performed satisfactorily in predicting adulteration at high concentrations (cross-validated coefficient of determination, *R_CV_*^2^, 0.95; standard error of cross-validation, *SECV*, 0.35% *v*/*v*). However, it demonstrated suboptimal performance in predicting adulteration at low concentrations.

### 2.4. Application of Artificial Intelligence Algorithms in Liquor Analysis

**Figure 2:**
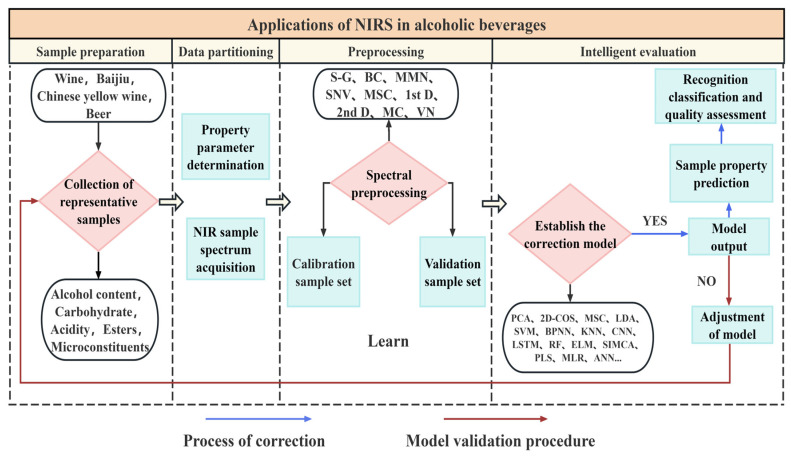
Diagrammatic representation of the procedure for employing near-infrared spectroscopy in the assessment of wine quality.

In recent years, models such as deep learning, convolutional neural networks, and Recurrent Neural Network (RNN) have improved the accuracy of classification and prediction. Wang et al. [35] combined SNV-SG preprocessing, UVE feature screening, and CNN to achieve the rapid detection of acidity in wine grains. Harris et al. [36] used an ANN-based NIR model to predict beer fermentation type (99% accuracy) and aroma composition (*R* > 0.92). Zhou et al. [37] constructed discriminative models using SVM, (Stacked Autoencoder, SAE) and 1D-CNN, and the accuracy of the SAE model was as high as 100% and 94%, which is instructive for the development of wine production and optoelectronic instruments. In addition, AI algorithms have the capacity for self-learning and optimization and can continuously update models based on new spectral data to maintain the stability of and improve their predictive performance. Through the introduction of AI algorithms, the field of wine analysis has been able to move to a higher level, bringing unprecedented opportunities for wine quality assessment. Figure 2 shows the application of NIR spectroscopy in the field of alcohol products.

## 3. Specific Applications of Near-Infrared Spectroscopy Combined with Chemometrics in Wine Quality Assessment

### 3.1. Rapid Determination of Alcohol and Accuracy Assessment

In the brewing industry, the rapid and non-destructive determination of alcohol concentration is of crucial importance. The combination of NIR spectroscopy and chemometrics provides an efficient solution strategy to meet this need. Menozzi et al. [38] examined ethanol and total acidity in fermented grape musts by employing traditional laboratory analytical methods with Fourier Transform Near-Infrared (FT-NIR) spectroscopy. The results are summarized in Table 1. The constructed predictive PLS model showed better performance in the 950–1650 nm band compared to the 800–1070 nm band. Liang et al. [39] used visible-near-infrared spectroscopy and hyperspectral imaging to analyze low-level fusion data after pretreatment and, combined with SVM-AdaBoost modeling, predicted the starch and alcohol content present during Baijiu grain fermentation. The XGBoost model for intermediate-level fusion data performed well in alcohol content prediction (*R_P_*^2^ = 0.9969, *RMSEP* = 0.0605), but its stability still needs to be further evaluated via cross-validation. Ana et al. [40] combined near-infrared spectroscopy with PLS to construct a multivariate calibration model for determining ethanol content in fermented alcoholic beverages. This PLS model yielded highly accurate analytical results with the advantages of easy operation, fast analysis, low cost, and environmental friendliness. Hu et al. [41] constructed a prediction model combining absorbance and alcohol concentration in the specific wavelength range of 1448 nm, and the effective range of this model covered the alcohol concentration range of 20% to 80% in the case of Baijiu. Meng et al. [42] used UVE (no information variable elimination), SPA (continuous projection algorithm), and PCA (principal component analysis) to screen the characteristic bands, and successfully reduced the spectral variables to 28, which significantly improved the prediction accuracy of white wine alcohol concentration, with a coefficient of determination (*R*^2^) of 0.9928. Luo et al. [43] combined the SPA with the iPLS model, which reduced the complexity of the model and improved the stability and accuracy, and this helped achieve the fast and non-destructive detection of yellow wine alcohol content. Chen et al. [44] used the Minimum Redundancy Maximum Relevance (mRMR) algorithm for the wavelength selection of NIRS to quantitatively detect the ethanol content in yellow wine, with *RPD* = 3.6875. Maskell et al. [45] assessed 218 kinds of beer samples to construct their model, but its generalization ability was affected by the complex matrix interference. Almeida et al. [46] conducted a comparative study on partial least squares (PLS), Monte Carlo uninformative variable elimination partial least squares (MCUVE-PLS), competitive adaptive reweighted sampling–partial least squares (CARS-PLS) and iterative information retained variable–partial least squares (iSPA-PLS). The results show that the iSPA-PLS-DA/OFF model performed well in Cachaça alcohol patients. Rimba et al. [47] measured the alcohol content in shochu moromi using NIRS, and the measurement error of NIRS was less than that exhibited by distillation oscillation densitometry. Therefore, the NIRS technique can be used to determine the alcohol content in sake mother in a simple and reliable way. The above findings provide solid technical support for the brewing industry, and help related companies to control the quality of their products more efficiently in order to meet consumer demand for high-quality alcoholic beverages.

### 3.2. Research on Quantitative Analysis Methods for the Contents of Major Components

**Figure 3:**
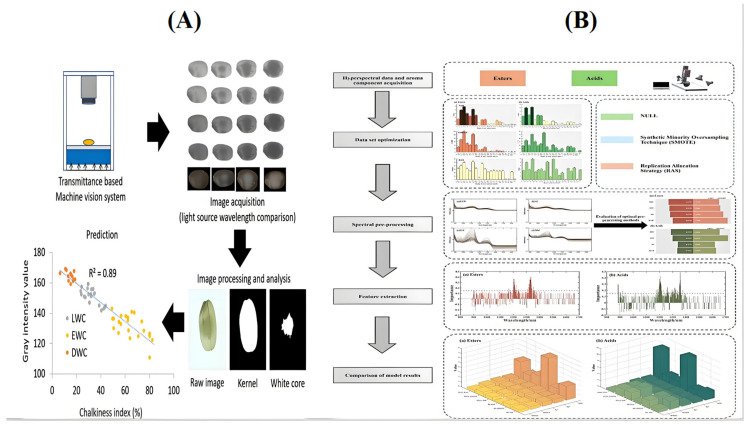
(**A**) The procedure for examining sake rice [49]. (**B**) Methodology for forecasting the aromatic constituents of Baijiu [54].

NIR spectroscopy has shown potential in quantitatively analyzing key chemical components of wine such as sugars, acids, esters, etc. Santos et al. [48] used NIR, MIR and Raman spectroscopy to quantitatively assess alcohol content, density, and total acidity in white wines. The *R_P_*^2^ values of NIR were 0.980 and 0.918 for alcohol content and density, respectively, which are higher than that of Raman spectroscopy. For total acid, volatile acid and pH, the *R_P_*^2^ < 0.75 and *RMSEP* > 10% of the NIR model were significantly higher than those of MIR and Raman spectroscopy, but may have been affected by the strong absorption of water and ethanol. Saha et al. [49] assessed the chalky white index of white-kerneled rice grains in a non-destructive manner by measuring the light transmission properties of rice in the visible to near-infrared region, and the correlation of the gray values with the chalky white index was high (*R*^2^ = 0.89). Therefore, the gray value of the NIR transmission image was sensitive to the chalky white index and suitable for sorting white kernels in the sake brewing industry. The procedure for checking sake rice is shown in Figure 3A. Lv et al. [50] combined partial least squares (PLS) with NIR quantitative models (correlation coefficients > 0.9) to establish a qualitative discriminant model for yellow wine. The near-infrared quantitative prediction model can accurately predict the content and color value of flavonoids, which provides a scientific basis for the quality identification of flavonoids during processing. Shen et al. [51] determined the routine quality parameters of Chinese yellow wine using NIR and MIR. By establishing a calibration model with full cross-validation, the partial least squares regression model performed best, and NIR outperformed MIR in pH (*R*^2^ = 0.797, *RPD* = 52.3) determination.

Kljusurić et al. [52] investigated the novel application of NIR spectroscopy integrated with stoichiometry for the non-destructive determination of parameters of ‘Maraština’ wine originating from Croatia’s Dalmatian region. The results demonstrate that NIR spectra, when combined with principal component regression analysis, effectively predicted the characteristics of Maraština wine, requiring only knowledge of grape juice attributes and NIR spectral data. This non-destructive methodology obviates the requirement for sample pretreatment, offering a straightforward and rapid approach to acquiring sample information. Zhu et al. [53] established a model for predicting the physicochemical indexes of fermentation spirits used in producing northern strong-flavored white wine by NIR, which was applicable to production analysis and testing, and the errors of the model were 0.77%, 0.11%, and 0.11% for entering spirits and 0.54%, 0.16%, and 0.49% for exiting spirits, respectively.

Huang et al. [54] combined NIR quantitative analysis and RF-RAS-SEL modeling, which combination performed well in the prediction of ester and acid content, with *R_P_*^2^ = 0.9803 and *RMSEP* = 0.3314 mg/L for ester content, and *R_P_*^2^ = 0.9914 and *RMSEP* = 0.4565 mg/L for acid content, indicating that the hyperspectral imaging technique can effectively and non-destructively detect esters and acids in liquor, providing a new approach to white wine aroma analysis, as shown in Figure 3B. Han et al. [55] used GC and FT-NIR for the simultaneous analysis of 3-methyl-1-butanol, 1-butanol and 1-propanol in Dukang-based wine. A calibration model was developed using PLS and evaluated by internal cross-validation. The FT-NIR spectral correction model has good predictive performance and high accuracy and can be used as an effective method for the rapid determination of alcohols in white wine. Su et al. [56] developed a rapid analytical model for ethyl acetate in raw wine, which functions by scanning the raw wine with NIR in combination with traditional analytical methods. The *R*^2^ values of the models all exceeded 0.95, and the values of the internal parameters *RMSEC* and *RMSEP* were similar and small, indicating the high quality of the models. External validation showed that the average relative error of the model prediction was 2.8%, which value is not significantly different from the traditional method and renders the approach suitable for practical production, providing an accurate quality control tool for wine producers. Liao et al. [57] used gas chromatography–mass spectrometry (GC-MS), combined with the MSC, CARS, and SVR methods, to detect volatile flavor substances in different grades of raw wines. The values of *R*^2^ = 0.8951 and *RMSE* = 0.03 were derived for the 17 principal components extracted by PCA. The accuracy, precision, and recall of the constructed model reached 99.10%, 99.62%, and 99.78%, respectively. The above results ensure the rapid and accurate prediction of the contents of key ingredients such as sugar, esters and organic acids via PLS, PCR and other modeling methods.

### 3.3. Qualitative Identification Techniques for Trace Ingredients and Flavor Substances

**Figure 4:**
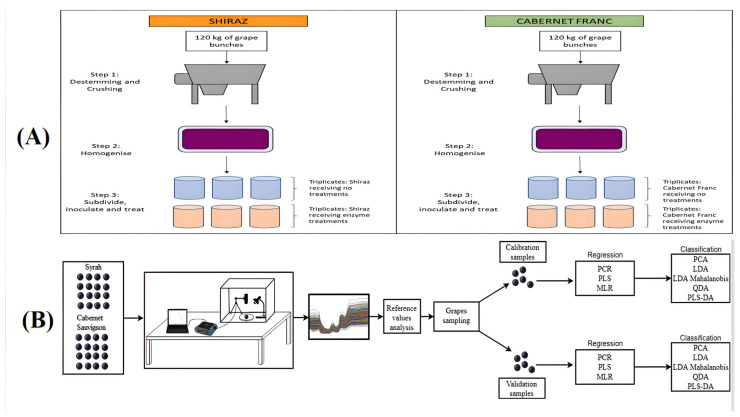
(**A**) The process of must crushing, destemming and division [58]. (**B**) The process used for developing mathematical regression and classification models for wine [63].

The qualitative identification of trace components and flavor substances is crucial when assessing the quality of wine. These components, although low in content, significantly affect the flavor and quality of wine. A combination of NIR spectroscopic techniques and chemometric methods can efficiently and accurately identify these components. Lambrecht et al. [58] investigated the effectiveness of T-FT-NIR, DF-FT-NIR, and ATR-FT-MIR for use in the removal of suspended solids during the fermentation of red wines, finding them to be suitable for online applications, while the spectral data and algorithmic modeling employed allowed for the precise identification and relatively accurate quantification of trace components (Figure 4A). Manzano et al. [59] developed a calibrated model using gas chromatography–mass spectrometry to analyze the volatile compounds in wines and the UV-Vis–NIR spectra of samples. The best calibration was achieved for the spectra of wine samples collected using a 1 mm cuvette, especially for decanoic acid (*r_v_*^2^ = 0.82; *RPD* = 3.37), ethyl caproate (*r_v_*^2^ = 0.94; *RPD* = 3.32), isoamyl acetate (*r_v_*^2^ = 0.89; *RPD* = 4.06), and phenyl ethyl acetate (*r_v_*^2^ = 0.95; *RPD* = 4.38). The results demonstrate the importance of optical range and its effects on the UV-Vis–NIR spectra of wine samples used for volatile compounds calibration.

In wine flavor analysis, olsmell measurement technologies (such as the electronic nose) capture volatile organic compounds (VOCs) by mimicking the human olfactory system, providing flavor fingerprints directly linked to sensory attributes. Chemometrics algorithms (such as PLS-DA and random forest) are responsible for decoding complex signals and establishing the quantitative relationship between VOCs and sensory indicators. The combination of the two can redress the subjective limitations of traditional sensory evaluations. Pettinelli et al. [60] investigated the fermentation of dehydrated grapes into passito wines and the addition of ethanol to produce fortified wines. Wine differences were rapidly detected using Near-Infrared–Acousto-Optic Tunable Filters (NIR-AOTF) and electronic nose techniques. At the end of dehydration, the sugars and total extracts increased significantly, with no significant differences between the two methods. Sweet wines had high concentrations of alcohols, esters, terpenes, and ketones and low concentrations of aldehydes; organic acids were concentrated but more easily oxidized. The electronic nose and NIR-AOTF were able to differentiate between sweet and fortified wines, but the electronic nose was limited in differentiating between different dehydration conditions. Bai et al. [61] analyzed wine samples using FT-NIR. Min-Max Normalization (MMN), PCA and Si-PLSR were used for data preprocessing, outlier removal, spectral characterization and modeling. The PLSR method resulted in a quantitative detection model with a coefficient of determination of 0.84 and *RMSECV* of 2.72. Véstia et al. [9] developed a calcium prediction model using FT-NIR for the rapid detection of calcium in grape juice and base wine. It was found that the optimal pretreatment method for base wine was baseline correction plus first-order derivatives, and the prediction model yielded *R*^2^ = 0.956, *RMSEP* = 3.05 mg/L, and *RPD* = 5.19. The FT-NIR technique was shown to offer an alternative to the AAS technique for assessing calcium content in grape must, which helps to prevent the blending of grape musts with high calcium contents. Cayuela et al. [62] investigated the feasibility of using visible/NIR spectroscopy techniques for assessing the sensory attributes of wines, and developed a prediction model for the good and bad sensory attributes of red and white wines. The technique accurately predicted key sensory attributes and defects of wines, but the accuracy of the prediction of flavor attributes of white wines was low, and the further optimization of sample diversity is needed.

Costa et al. [63] collected spectral data from “Syrah” and “Cabernet Sauvignon” grapes, and developed an MLR prediction model using PCR and PLSR, combining full spectral data with a selected set of spectral samples to provide reliable predictions of total soluble solids and anthocyanin content (*R*^2^ ≥ 0.90) (Figure 4B). Fernández et al. [64] explored the potential application of NIR in the detection of beer gluten, and compared the results of different ELISA kits. The NIR values of the whole samples were correlated with the ELISA value (*R*^2^ = 0.167), but the complex components (e.g., alcohols, polyphenols) produced by the fermentation process interfered with the spectral signals, which led to the inability of NIR to accurately model the results. Quoc et al. [65] used NIR to detect and validate the methanol content in ethanol-doped methanol and Vietnamese vodka samples. Ethanol and alcoholic beverage samples with 5% variations in methanol contents were utilized to validate the prediction algorithm. The SNV algorithm was applied to calibrate the NIR spectra, and was successfully used to classify alcohol adulteration, with 98% accuracy. In the study of the qualitative identification of alcohol trace components and flavor substances, NIR spectroscopy combined with chemometrics breaks through the limitations of traditional detection techniques. Feature information extraction and pattern recognition were performed using PCA and other methods, and when this was combined with intelligent algorithms such as SVM and ANN, trace substances such as aldehydes, phenols, terpenes, etc., and their flavor contributions could be effectively identified. Table 1 below shows the results of the application of NIR spectroscopy in the detection of the chemical composition of alcohol.

**Table 1:** NIRS used for the chemical composition analysis of liquor.

Detection Object	Instrument Type	Preprocessing Method	Band Range	Modeling Approach	Accuracy (%) Result	Reference
Wine	FT-NIR	S-G + SNV + MC	4000–12,500 cm^−1^	PCA + PLS	*R_P_*^2^ = 0.990	[38]
Baijiu	NIR	SNV + MSC + S-G	886–1735 nm	PLSR + SVM + LLF + ILF	*R_P_*^2^ = 0.9925	[39]
Liquor	NIR	S-G + SNV + MC	908–1676 nm	PLS	*RPD* = 4.1	[40]
Baijiu	UV-Vis-NIR	SNV + MSC	1000–1800 nm	PLS	*R*^2^ = 0.9993	[41]
Baijiu	NIR	UVE + SPA + PCA	770–2500 nm	PLS	*R*^2^ = 0.9928	[42]
Baijiu	FT-NIR	SPA + iPLS	770–2500 nm	PLSR	*R*^2^ = 0.952	[43]
Chinese yellow wine	NIR	-	770–2500 nm	PLSR + SVR	*R* = 0.9848 *RPD* = 3.6875	[44]
Beer	NIR	-	800–2500 nm	-	*RMSE* = ±0.33% *v*/*v*	[45]
Cachaca	NIR	OFF + SGD	1350–1850 nm	PLS	*RPD* = 59.04 *R_P_* = 0.99	[46]
Shochu	NIR	-	780–2500 nm	LR	*R* = 0.99	[47]
Wine	NIR	S-G + SNV	1100–2498 nm	PLS	*R_P_*^2^ = 0.98	[48]
Sake	Vis-NIR	GLM	350–1800 nm	LSM	*R*^2^ = 0.89	[49]
Chinese yellow wine	FT-NIR	MSC + S-G + SNV	3999–10,001 cm^−1^	PLS-DA	*R* = 0.9852	[50]
Chinese rice wine	FT-NIR	MSC + S-G	4000–12,000 cm^−1^	PLSR + SMLR + ANN	*R_V_*^2^ = 0.941 *RPD* = 4.1	[51]
Baijiu	NIR	MC + SNV + MSC	900–2500 nm	PLS + SPXY	*R*^2^ = 0.92	[53]
Baijiu	NIR	MSC + SNV + S-G	900–1700 nm	PSO-SVR + RBFNN + PLSR + SEL	*R_P_*^2^ = 0.9914 *RPD* = 10.8007	[54]
Base Liquor	FT-NIR	MSC	11,998–4597 cm^−1^	PLS	*R*^2^ = 97.96	[55]
Base Liquor	FT-NIR	MSC + SNV + PLS	780–2526 nm	PLS	*R*^2^ = 0.9694	[56]
Original liquor	FT-NIR	MSC + SNV + CARS	4000–12,500 cm^−1^	SVR + RF + PCA	100%	[57]
Red wine	FT-NIR	-	12,500–4000 cm^−1^	PLS + PCA	*R*^2^ = 0.9211	[58]
Wine	NIR	-	1100–2600 nm	PLS-DA	100%	[60]
Wine	FT-NIR	MMN + VN + FD + SD + PCA	870–2500 nm	PLSR + Si-PLS	*RPD* = 2.39	[61]
Base wine	FT-NIR	BC + S-G + MSC + SNV	1100–2300 nm	PLSR	*R*^2^ = 0.935 *RPD* = 4.39	[9]
Wine	NIR	MC + S-G	350–2500 nm	PLS	*R* = 0.92 *RPD* = 2.54	[62]
Wine	NIR	MSC + SNV + S-G	450–1800 nm	PCR + PLSR + MLR + PLS + PLS-DA	*RPD* = 4.44	[63]
Beer	NIR	MSC	1400–2400 nm	MLR	*R*^2^ = 0.592	[64]
Vodka	NIR	-	700–1070 nm	SNV	98%	[65]

### 3.4. Identification and Traceability Techniques for Counterfeit Liquor

The use of NIR spectroscopy technology combined with chemometrics has been outstanding in the authentication and tracing of wine. By collecting NIR spectral data from counterfeit wines and applying models such as PLSR, LDA, ANN, and RF, researchers have successfully constructed a high-precision identification system with an accuracy rate often more than 95%. Su et al. [66] constructed a wine traceability model based on phenolics and FTIR spectroscopy, and the LDA and ANN outperformed the PLS-DA and RF. Machyňáková et al. [67] achieved a 100% accurate classification of Slovakian wines using the DD-SIMCA model, with good predictive performance shown by the PLS model (*R*^2^ = 0.95, *RMSEP* = 0.44). Harris et al. [68] analyzed closed bottles of wine by NIR and accurately predicted the vintage year (97.2%) and organoleptic characteristics (*R* = 0.95). Yu et al. [69] used NIR, RBFNN and LS-SVM to identify wines of different varieties. It was found that the RBFNN model with SNV preprocessing and the LS-SVM model with MSC preprocessing achieved a classification accuracy of 98.36%. However, advanced algorithms such as random forest and deep learning were not included in the performance comparison, and the optimality of the models has not been verified.

For white wine, Han et al. [70] investigated the feasibility of using FT-NIR for the rapid determination of ethyl valerate in Chinese Baijiu. A calibration model for the prediction of ethyl valerate content was established using partial least squares, and the results yielded values of *R*^2^ = 0.964 and *RMSEP* = 0.023 g/L. Miao et al. [19] fused UV, NIR and multidirectional fluorescence spectra, and combined them with intermediate data fusion techniques to achieve a nearly 100% accurate identification of white wine. Zong et al. [71] detected the year of the base wine based on LSSVM and the grade, but the *R*^2^ was low (56.72%, 58.17%), leaving room for optimization. Gonzalez et al. [72,73] successfully classified 85% of the fermentation types using ANN models based on NIR spectroscopy to predict the pH and alcohol content of beer. Hu et al. [74] utilized benchtop and micro NIR to identify the origin of yellow wine with 100% accuracy. Peng et al. [75] combined FA and PLS models to detect the adulteration of glutinous rice in yellow wine, and the *R*^2^ was over 98%. Jakubíková et al. [76] explored the feasibility of using Synchronous Fluorescence Spectroscopy (SFS), UV-Vis and NIR combined with chemometrics to identify plum brandy from different varietal origins, demonstrating its potential in spite of the limited prediction accuracy (66%). Overall, spectroscopy combined with modeling methods is effective in improving the efficiency of the authenticity identification and tracing of alcohol, but the portability of the equipment, the online monitoring capacity and the model generalization ability still need to be improved. Table 2 below shows the application of NIR spectroscopy in the identification and tracing of alcohol products.

### 3.5. Methods for the Dynamic Monitoring of Quality Changes in the Aging Process

NIR spectroscopy combined with chemometrics techniques provides a powerful tool for the real-time monitoring of quality changes during wine storage and fermentation. By regularly collecting spectra and applying methods such as PCA and PLS, dynamic changes in alcohol content, phenolics, and other components can be accurately tracked, and future quality trends can be predicted via combination with machine learning models. Littarru et al. [77] employed near-infrared spectroscopy and electronic nose modeling to accurately predict the contents of polyphenols, anthocyanins, glucose and fructose in wine. Alfieri et al. [78] showed that low-cost near-infrared equipment can monitor phenolic compound extraction during wine fermentation via real-time, non-destructive measurements. While less predictive than commercial FT-NIR systems, the prototype exhibited good accuracy and could offer an economically efficient alternative for wineries desiring cost-effective phenolic analysis after optimization. Vann et al. [79] used near-infrared online monitoring technology to monitor the fermentation indicators TCC (total cell concentration), SG (specific gravity), FAN (free amino nitrogen) and % V-1 (volume percentage) in beer in real time, so as to support the implementation of feedforward control strategies, and thus regulate yeast inoculum usage and improve fermentation stability. Hua [80] and Wang [81], among others, demonstrated that FT-NIR, in combination with iPLS or PLSR, can rapidly predict the polyphenol, residual sugar, and alcohol concentrations in fruit wine (Kiwi wine) fermentation, but many studies still lack comprehensive considerations of other key indicators.

**Figure 5:**
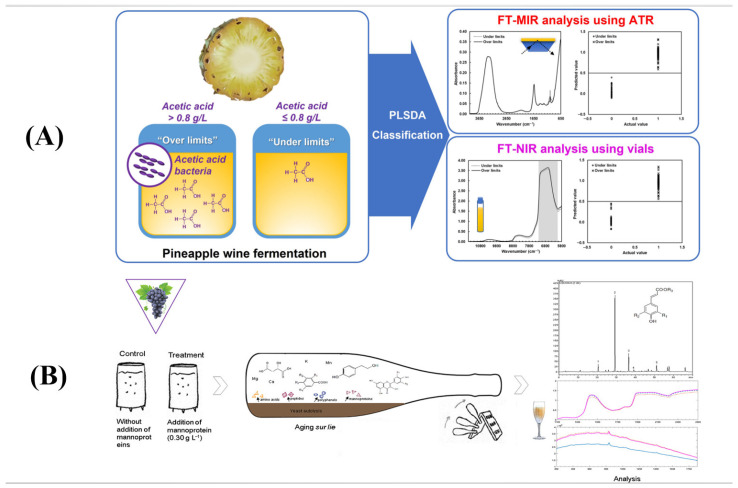
(**A**) NIR for the monitoring and classification of the fermentation process of pineapple wine [82]. (**B**) NIR for monitoring the aging process of rosé sparkling wine [83].

Kasemsumran et al. [82] investigated the classification of fermentation anomalies in pineapple wine using MIR and NIR (Figure 5A). Sartor et al. [83] explored the effects of mannoproteins on the evolution of quality in rosé sparkling wine during the aging process of the mash, assessing the effect of NIR and Raman spectroscopy. The dynamic changes in polyphenolics, organic acids, and macro/trace elements were evaluated, demonstrating the potential of vibrational spectroscopic processing (Figure 5B). Aleixandre et al. [84] evaluated the efficacy of the FT-NIR technique in the quantitative analysis of phenolic compounds in wine using FT-NIR. The study showed that the relative performance diagnostic (*RPD*) values of the FT-NIR technique when used on five phenolic compounds, including monomeric condensed tannins (MCPs) and total phenolic indices (TPIs), were greater than 3, suggesting that the technique is suitable for real-time monitoring. Hu et al. [85], on the other hand, classified Cabernet Sauvignon wines from different production regions by means of near-infrared and mid-infrared spectroscopy, and the application of this technique not only helped to improve the storage quality of wine products but also offers novel approaches to the sustainable development of the wine industry. In summary, Table 3 shows the application of NIR spectroscopy in monitoring different alcohol products during storage.

**Table 3:** NIRS for monitoring liquor during storage.

Detection Object	Instrument Type	Preprocessing Method	Band Range	Modeling Approach	Accuracies (%)	Reference
Wine	NIR	PCA	1100–2300 nm	PCR	*R_CV_*^2^ = 0.68	[77]
Wine	NIR	S-G	1350–2150 nm	PLS	*RPD* = 3.03 *R*^2^ = 0.91	[78]
Fruit wine (kiwi wine)	FT-NIR	VN	6101–5446 cm^−1^	PLS	*R_P_*^2^ = 0.978	[81]
Fruit wine (Pineapple wine)	FT-NIR	SD + SNV	11,536–3952 cm^−1^	PLS-DA	100%	[82]
Rosé sparkling wine	FT-NIR	PCA	9091–4000 cm^−1^	PCA	100%	[83]
Wine	FT-NIR	MSC + MMN	10,500–4300 cm^−1^	PLS	*RMSEP* = 71.1 mg/L *R*^2^ = 0.97	[84]
Wine	FT-NIR	SNV	4555–4353 cm^−1^	PCA + SIMCA + DA	97%	[85]

## 4. Technical Challenges and Trends in Near-Infrared Spectroscopy Combined with Chemometrics

### 4.1. Research on Effective Elimination Methods for Complex Matrix Interference

NIR spectroscopy encounters complex matrix interferences during the analysis of alcoholic beverages. To address this, researchers have employed chemometric techniques such as multiple scattering correction, Principal Component Analysis (PCA), and Partial Least Squares (PLS) to purify the data. Helfer et al. [86] utilized a Raspberry Pi cluster to process data from 60 beer samples, achieving a model fit of 0.999 and a Root Mean Square Error of Cross-Validation (*RMSECV*) of 0.216, thereby enhancing processing efficiency. Molla et al. [87] conducted an investigation into the influence of diverse path lengths on the modeling of phenolic compounds within wine. The study revealed a discernible impact on the Standard Error of Cross-Validation (*SECV*); however, no significant variance was identified. This outcome underscores the need for the meticulous consideration of light scattering errors. Ling et al. [88] developed an RPLS update model, which leveraged characteristic wavelengths to adapt to fluctuations in raw materials and environmental conditions, thereby enhancing the stability of yellow wine detection. The model demonstrated a correlation coefficient (*r*) of 0.9657, an *RMSEP* of 0.1843, and an *RPD* of 3.7362. This approach not only optimized the stability of the model, but also augmented computational efficiency and offered a pragmatic reference. Debebe et al. [89] integrated preprocessing techniques with PLS modeling to precisely ascertain the ethanol and methanol contents in distilled spirits, with the predicted outcomes aligning with those obtained through gas chromatography. Looking ahead, the integration of machine learning and deep learning methodologies is anticipated to facilitate the more efficient elimination of interferences within complex matrices, thereby enhancing the precision and intelligence of alcohol spectral analysis.

### 4.2. Current Status of Development and Application of Online Inspection Systems

In contexts requiring high accuracy and legal compliance, benchtop Fourier near-infrared spectroscopy remains indispensable. Its principal advantage resides in generating traceable and precise data to satisfy quality certification system requirements. Nevertheless, low-cost portable near-infrared spectrometers (NIRs) demonstrate irreplaceable value for dynamic process monitoring and real-time decision-making in alcohol production. Their core capability involves transferring laboratory functionalities to production facilities, thereby addressing latency issues inherent in traditional analytical methods. Furthermore, to mitigate inter-instrument discrepancies, model transfer between primary and secondary instruments can be implemented.

The implementation of an online detection system presents an innovative solution to the challenge of complex matrix interference in the evaluation of wine quality. This system is equipped with the ability to gather and process spectral data in real time, and through the application of sophisticated algorithms, it can automatically rectify interferences, thereby enhancing the precision and efficiency of analysis. It has found extensive application in both production processes and quality control. Fulgêncio et al. [90] engineered a portable NIR device coupled with PLS-DA, enabling the rapid detection of diethylene glycol (DEG) in beer samples with a high degree of accuracy and without the need for reagents or solvents, making it appropriate for quality control or forensic analysis. Franca et al. [91] developed an online detection system that is also appropriate for quality control or forensic analysis. The outcomes indicate that using partial least squares regression for monitoring the soluble content of solids (SSC) is a method characterized by its versatility, simplicity, and reliability, and is suitable for the comprehensive monitoring and control of the beer production process. Aleixandre et al. [92] utilized a non-contact FT-NIR instrument to explore the feasibility of the online monitoring of phenolics in red wine grapes. The model’s predictive accuracy and errors in relative analysis for tannins, anthocyanins, total phenols, and color density were 12% and 2.37, 12.3% and 3.37, 7.8% and 3.2, and 16.7% and 2.84, respectively. Rouxinol et al. [93] used a portable NIR spectrometer to quantitatively analyze the soluble solids content (SSC), titrable acidity (TA), total phenolic content, total flavonoids, total anthocyanins and total tannins in four red wine grape varieties, using a model constructed by a partial least squares regression algorithm. The determination coefficients of SSC, TA, total anthocyanins and total tannins were 0.89, 0.90, 0.87 and 0.88, respectively. The residual prediction bias (*RPD*) values of these models were all higher than 2.3, which could provide a theoretical basis for the subsequent quantification of quality indicators in wine grapes. However, the acquisition of spectral data was consistently time-intensive, hindering the collection of real-time feedback from the production line. In summary, ongoing technological innovation and system optimization are fundamental to the widespread adoption of in-line inspection systems within the domain of wine quality assessment.

### 4.3. Synergistic Analysis Strategy with Multi-Technology Linkage

The combined utilization of NIR spectroscopy, gas chromatography–mass spectrometry (GC-MS), and High-Performance Liquid Chromatography (HPLC) techniques not only facilitates the identification of principal components within alcoholic beverages, but also elucidates the interrelationships among these components, offering a holistic approach to quality evaluation. This approach surmounts the constraints inherent to individual methodologies and enhances the dependability and precision of the outcomes.

Liu et al. [94] integrated FT-NIR spectroscopy with gas chromatography (GC) to develop a PLS model for the quantification of ethyl caprylate in white wine, achieving an optimal result with a coefficient of determination (*R*^2^) of 0.9507 and a Root Mean Square Error of Cross-Validation (*RMSECV*) of 3.91 mg/L, based on the Standard Normal Variate (SNV) preprocessing method. Anjos et al. [95] combined Gas Chromatography–Flame Ionization Detector (GC-FID) with NIR spectroscopy to examine the alterations in volatile components of wine spirits resulting from aging in various containers over different periods, as well as the concentrations of alcoholic and volatile components such as methanol and acetaldehyde. The NIR technique has demonstrated the ability to discern the impacts of distinct aging processes and types of wood on the aging characteristics. Tzanova et al. [96] utilized High-Performance Liquid Chromatography in conjunction with ultraviolet and near-infrared (HPLC-UV-NIR) detection to ascertain the active components (resveratrol, quercetin, and total phenolic content) and the antioxidant capacities of wines, achieving a correlation coefficient (*R*) greater than 0.97 and an *RPD* exceeding 4. Ripoll et al. [97] have utilized ultraviolet–visible–near-infrared (UV-VIS-NIR) spectroscopy to rapidly predict the concentrations of volatile compounds such as (E)-2-hexenal and 1-hexanol in Albariño grapes, with an *R*^2^ greater than 0.85, indicating the robust predictive capability of the model. Hanousek et al. [98] employed NIR spectroscopy in conjunction with PCA/PLS to differentiate among various aged liquors, achieving a classification accuracy of 100% in phenolic-based categorization. With the ongoing advancement of technology, the synergistic analytical approach, characterized by the integrated application of multiple techniques, will assume an increasingly vital role in the assessment of wine quality.

### 4.4. Prospects for the Application of Artificial Intelligence and Big Data Technologies in Model Optimization

Artificial Intelligence and Big Data technologies show great potential for use in the optimization of wine quality assessment models combining NIR spectroscopy and chemometrics. Integrating machine learning algorithms such as deep learning and neural networks significantly improves the prediction accuracy and generalization ability of the models. These algorithms can automatically extract complex features and more accurately explain the relationship between alcohol composition and quality. Big data technology provides technical support for model optimization by analyzing a large amount of sample data to construct richer training sets and enhance the robustness and applicability of the models. Viejo et al. [99] constructed an AI model based on aroma profiles and NIR data to predict beer aroma, with an *R* of 0.91 to 0.93. Yan et al. [100] employed collapsed PCA + SVM/DNN to attain a precision exceeding 99% in the non-destructive identification of phenolics. Hu et al. [101] introduced the ACO algorithm for wavelength selection, which resulted in PLSR modeling accuracies that were significantly superior to those of full-spectrum or traditional selection methods. Fuentes et al. [102] used ANN to analyze wine NIR data across vintages, and this approach excelled in predicting sensory profile (*R* = 0.98) and color (*R* = 0.99).

CNN and RNN models achieve high accuracy in spectral tasks, but their deployment faces key limitations. First, supervised learning requires large labeled datasets, yet obtaining accurate wine spectral labels with tools like GC-MS is costly. Second, performance fluctuates across wines coming from different origins, vintages, or fermentation processes. Third, CNN extracts features, but identifying key wavelengths is challenging. Finally, data disparities between different spectrometers degrade models. Additionally, open wine or spirits databases are scarce, focus on basic components and lack deep spectral data. Therefore, to advance the use of AI in alcohol spectral analysis, future wine–lab collaboration is needed to encourage the sharing of spectral data, establish alcohol databases, and embed models into portable devices for rapid determination. Looking forward, the integration of artificial intelligence and big data technologies will further promote the application of NIR spectroscopy and chemometrics in the field of wine quality assessment. It is expected that these advanced technologies can provide us with more accurate, efficient and intelligent methods for wine quality assessment, and contribute more to the sustainable development of the wine industry.

## 5. Summary and Outlook

NIR spectroscopy, in conjunction with chemometric techniques, has transitioned from theoretical exploration to practical application in the evaluation of wine quality. This advancement is characterized by its rapidity, non-invasive nature, and precision in detection. Consequently, this technology is progressively emerging as a pivotal instrument for quality assurance in wine production, the development of new products, and market oversight. Nevertheless, challenges such as intricate matrix interferences, inadequate stability in online systems, and complexities associated with the integration of multiple technologies continue to impede its progression. At the same time, it is necessary to further explore the highly sensitive detection of trace risk substances (such as biogenic amines and mycotoxins) in the complex matrix of wine [103]. By combining multispectral techniques with deep feature extraction algorithms, the ability to capture risk factors at low concentrations can be improved, providing a double guarantee for wine safety. Future scholarly endeavors should concentrate on the following:

(1) Augmenting investigations into the mechanisms underlying complex matrix interferences and delving into more sophisticated data preprocessing methodologies and feature extraction techniques to bolster the model’s resilience against such interferences.
(2) Enhancing the architecture and algorithms of online detection systems to elevate the system’s stability and detection precision, thereby fulfilling the requisite for swift detection in real-world production scenarios.
(3) Intensifying research on synergistic technology use for precise wine quality evaluation.
(4) Incorporating AI and Big Data analytics via machine learning to enhance model accuracy.
(5) Uncovering deeper spectral data–quality metric correlations to expand the informational scope.

The fusion of near-infrared spectroscopy and chemometrics holds promise, and sustained innovation and tech integration foster collaborative advancements in academia and industry. Most of the existing literature reviews concentrate on single-technology analyses, while systematic discussions incorporating multi-technology collaborative analytical frameworks are scarce. The international review literature has not sufficiently addressed the spectral characteristics of traditional Chinese alcoholic beverages, such as Baijiu and Huangjiu, nor their corresponding industrial technical bottlenecks. In view of this, this paper proposes the concept of establishing a near-infrared (NIR) detection standard framework for alcoholic products in China, aiming to facilitate industry standardization development.

## Figures and Tables

**Table 2:** NIRS used for identification and traceability of liquor.

Detection Object	Instrument Type	Preprocessing Method	Band Range	Modeling Approach	Accuracies (%)	Reference
Wine	NIR	-	1596–2396 nm	ANN	*R* = 0.92	[66]
Wine	NIR	PLS + S-G	4000–650 cm^−1^	DD-SIMCA	100%	[67]
Wine	NIR	SGD + OFF	1596–2396 nm	ANN	*R* = 0.95	[68]
Wine	FT-NIR	SNV + MSC + PCA	1000–2500 nm	RBFNN	100%	[69]
Baijiu	FT-NIR	MSC	6105–5446 cm^−1^	PLS	*R*^2^ = 0.964 *RMSEP* = 0.023 g/L	[70]
Baijiu	NIR	SNV + MSC	4833–6846 cm^−1^	LSSVM	*R_P_*^2^ = 58.17% *RMSEP* = 0.2134 *RPD* = 44.72	[71]
Beer	NIR	-	1600–2396 nm	PLS + ANN	*R*^2^ = 0.99	[72]
Beer	NIR	S-G	1596–2396 nm	ANN	*R* = 0.99	[73]
Chinese rice Wine	NIR	VN + S-G + SNV	833–2500 nm	FA + PCA	*R*^2^ = 0.988	[74]
Chinese rice Wine	FT-NIR	SNV	10,828–3949 cm^−1^	FA + PLS	*R_P_*^2^ = 98.3% *RMSEP* = 4.2%	[75]
Brandies	FT-NIR	PCA	4000–7500 cm^−1^	PCA + LDA	100%	[76]

## Data Availability

Not applicable.

## References

[1] Liu C.Y., Yan T.Y., Fu T.T., Wang K., Rong X.D., Liu X.T., Wang Y., Cai X.Y., Sheng W.L., Zhu B.C. (2025). A NIR fluorescent probe based on carbamoyl oxime with high specificity for detecting ferrous ions in food and in vivo. Food Res. Int..

[2] Xu X.J., Zeng Y.L., Ding H.C., Liu Q.L., Mao L.T., Liu G., Pu S.Z. (2024). Rapidly responsive and highly selective NIR fluorescent probe for detecting hydrogen sulfide in food samples and living cells. Spectrochim. Acta Part A Mol. Biomol. Spectrosc..

[3] Liu J., Munnaf M.A., Mouazen A.M. (2024). Micro-near-infrared (micro-NIR) sensor for predicting organic carbon and clay contents in agricultural soil. Soil Tillage Res..

[4] Zhao A.Y., Fu X.P., Wu J.Q., Zhang J.Y. (2025). Calibration transfer of sugar content prediction models for agricultural products via NIR spectral augmentation and reconstruction architecture. Biosyst. Eng..

[5] Wang Z.Q., Wan X.H., Luo X.R., Yang M., Wang X.C., Zhong Z.J., Tao Q., Wu Z.F. (2025). Development of a Data Fusion Strategy Combining FT-NIR and Vis/NIR-HSI for Non-Destructive Prediction of Critical Quality Attributes in Traditional Chinese Medicine Particles. Vib. Spectrosc..

[6] Chen Q.T., Li J.X., Wang Y.T., Tian M.Y., Liang T.Y., Zhong K.L., Yan X.M., Tang L.J. (2025). A quinolinium-based colorimetric and NIR fluorescent dual-channel sensing platform for specific detection of bisulfite in food, traditional Chinese medicine and living cells. Dye. Pigment..

[7] Jakabová S., Fikselová M., Mendelová A., Ševčík M., Jakab I., Aláčová Z., Kolačkovská J., Ivanova-Petropulos V. (2021). Chemical Composition of White Wines Produced from Different Grape Varieties and Wine Regions in Slovakia. Appl. Sci..

[8] Ferrara G., Melle A., Marcotuli V., Botturi D., Fawole O.A., Mazzeo A. (2022). The prediction of ripening parameters in Primitivo wine grape cultivar using a portable NIR device. J. Food Compos. Anal..

[9] Véstia J., Barroso J.M., Ferreira H., Gaspar L., Rato A. (2019). Predicting calcium in grape must and base wine by FT-NIR spectroscopy. Food. Chem..

[10] Chen K., Wang S.W., Liu S.H. (2025). Wine composition detection utilizing 1D-CNN and the self-attention mechanism. Vib. Spectrosc..

[11] Chen H., Tan C., Lin Z. (2025). Application of subspace ensemble radical basis function networks to quantitative analysis of near-infrared and mid-infrared spectroscopy. Microchem. J..

[12] Yu S., Huan K.W., Liu X.X., Wang L., Cao X.W. (2023). Quantitative model of near infrared spectroscopy based on pretreatment combined with parallel convolution neural network. Infrared Phys. Technol..

[13] Huang Y.X., Tian J.P., Yang H.L., Hu X.J., Xie L.L., Zhou Y.F., Xia Y.Y., Huang D. (2024). Utilization of hyperspectral imaging for the analysis of aroma components of Soy Sauce-Aroma Type Baijiu. J. Food Compos. Anal..

[14] Ríos-Reina R., Segura-Borrego M.P., Camiña J.M., Callejón R.M., Azcarate S.M. (2025). Multiplatform spectralprint strategies for the authentication of Spanish PDO fortified wines using AHIMBU, an automatic hierarchical classification tool. Chemom. Intell. Lab. Syst..

[15] Zhou X.J., Li L., Zheng J., Wu J.H., Wen L., Huang M., Ao F., Luo W.L., Li M., Wang H. (2024). Quantitative analysis of key components in Qingke beer brewing process by multispectral analysis combined with chemometrics. Food Chem..

[16] Cavallini N., Cavallini E., Savorani F. (2025). Monitoring the homemade fermentation of readymade malt extract using the SCiO NIR sensor, A convergence of technology and tradition. Spectrochim. Acta Part A Mol. Biomol. Spectrosc..

[17] Bai H.J., Li Y., Wu Y.Q. (2023). Insights into ethanol–water clusters in alcoholic beverages by vibration spectroscopy connecting with quality and taste. J. Mol. Liq..

[18] Jin X., Wu S., Yu W., Xu X., Huang M., Tang Y., Yang Z. (2019). Wine Authentication Using Integration Assay of MIR, NIR, E-tongue, HS-SPME-GC-MS, and Multivariate Analyses: A Case Study for a Typical Cabernet Sauvignon Wine. J. AOAC Int..

[19] Miao H., Xiao L.C., Jing Z., Li J.W., Zhao D., Huang Y., Huo D.Q., Luo X.G., Hou C.J. (2023). Identification of liquors from the same brand based on ultraviolet, near-infrared and fluorescence spectroscopy combined with chemometrics. Food Chem..

[20] Ouyang Q., Chen Q., Zhao J. (2016). Intelligent sensing sensory quality of Chinese rice wine using near infrared spectroscopy and nonlinear tools. Spectrochim. Acta Part A Mol. Biomol. Spectrosc..

[21] Bianchi A., Pacifico S., Santini G., Pettinelli S., Alfieri G., Modesti M., Bellincontro A., Sanmartin C., Pittari E., Piccolella S. (2025). Carbonic or nitrogen maceration of wine grape: Biochemical differences of grape and wine using destructive and non-destructive approach. Food Chem..

[22] Welke J.E., Hernandes K.C., Lago L.O., Silveira R.D., Marques A.T.B., Zini C.A. (2024). Flavoromic analysis of wines using gas chromatography, mass spectrometry and sensory techniques. J. Chromatogr..

[23] Zhang C.J., Liang Y.Y., Du W.H., Kuang M.M., Meng Z.Y., Gong S., Wang Z.L., Wang S.F. (2024). A novel BODIPY-based colorimetric turn-on NIR fluorescent probe for sensitive and visual detection of H_2_S in food samples with smartphone platform. J. Food Compos. Anal..

[24] Joshi I., Truong V.K., Chapman J., Cozzolino D. (2020). The use of two-dimensional spectroscopy to interpret the effect of temperature on the near infrared spectra of whisky. J. Near Infrared Spectrosc..

[25] Wang X.F., He M., Zheng J., Ma Y., Luo H.B., Hou C.J., Huo D.Q. (2024). Methodology and optimization research for discrimination of different brands of Baijiu based on multispectral techniques. J. Food Meas. Charact..

[26] Zhang G.Y., Tuo X.G., Peng Y.J., Li X.P., Pang T.T. (2024). A Rapid Nondestructive Detection Method for Liquor Quality Analysis Using NIR Spectroscopy and Pattern Recognition. Appl. Sci..

[27] Wang Z.K., Ta N., Wei H.C., Wang J.H., Zhao J., Li M. (2024). Research of 2D-COS with metabolomics modifications through deep learning for traceability of wine. Sci. Rep..

[28] Pu S.S., Zheng E.R., Chen B. (2023). Research on a classification algorithm of near-infrared spectroscopy based on 1D-CNN. Spectrosc. Spectr. Anal..

[29] Zhang Z.T., Li Y., Bai L., Chen P., Jiang Y., Qi Y.L., Guan H.H., Liang Y.X., Yuan D.P., Lu T.L. (2024). Machine learning combined with multi-source data fusion for rapid quality assessment of yellow rice wine with different aging years. Microchem. J..

[30] Aiello G., Tosi D. (2024). An Artificial Intelligence-based tool to predict “unhealthy” wine and olive oil. J. Agric. Food Res..

[31] Koljančić N., Furdíková K., Araújo Gomes A., Špánik I. (2024). Wine authentication: Current progress and state of the art. Trends Food Sci. Technol..

[32] Harris N., Viejo C.G., Barnes C., Pang A., Fuentes S. (2023). Wine quality assessment for Shiraz vertical vintages based on digital technologies and machine learning modeling. Food Biosci..

[33] Silva R., Freitas O., Melo Pinto P. (2024). Evaluating the generalization ability of deep learning models: An application on sugar content estimation from hyperspectral images of wine grape berries. Expert. Syst. Appl..

[34] Kolobaric A., Orrell-Trigg R., Orloff S., Fraser V., Chapman J., Cozzolino D. (2023). The Use of a Droplet Collar Accessory Attached to a Portable near Infrared Instrument to Identify Methanol Contamination in Whisky. Sensors.

[35] Wang Q.B., He Y.K., Luo Y.S., Wang S.J., Xie B., Deng C., Liu Y., Tuo X.G. (2023). Study on Analysis Method of Distiller’s Grains Acidity Based on Convolutional Neural Network and Near Infrared Spectroscopy. Spectrosc. Spectr. Anal..

[36] Harris N., Gonzalez Viejo C., Zhang J.Y., Pang A., Hernandez-Brenes C., Fuentes S. (2025). Enhancing beer authentication, quality, and control assessment using non-invasive spectroscopy through bottle and machine learning modeling. J. Food Sci..

[37] Zhou X.J., Liu W.Z., Li K., Lu D.Q., Su Y., Ju Y.L., Fang Y.L., Yang J.H. (2023). Discrimination of maturity stages of cabernet sauvignon wine grapes using visible–near-infrared spectroscopy. Foods.

[38] Menozzi C., Foca G., Calvini R., Catellani L., Bezzecchi L., Ulrici A. (2024). Comparison of Different Spectral Ranges to Monitor Alcoholic and Acetic Fermentation of Red Grape Must Using FT-NIR Spectroscopy and PLS Regression. Food Anal. Methods.

[39] Liang Y., Tian J.P., Hu X.J., Huang Y.X., He K.L., Xie L.L., Yang H.L., Huang D., Zhou Y.F., Xia Y.Y. (2024). Rapid determination of starch and alcohol contents in fermented grains by hyperspectral imaging combined with data fusion techniques. J. Food Sci..

[40] da Costa Fulgêncio A.C., Resende G.A.P., Teixeira M.C.F., Botelho B.G., Sena M.M. (2023). Combining Portable NIR Spectroscopy and Multivariate Calibration for the Determination of Ethanol in Fermented Alcoholic Beverages by a Multi-Product Model. Talanta Open.

[41] Hu Y.Q., Guo M., Ye X.S., Li Q., Liu H.N., Wu Z.J. (2022). Indirect Determination of Liquor Alcohol Content Based on Near-Infrared Spectrophotometry. Spectrosc. Spectr. Anal..

[42] Zhao M., Sun A., He S.Y. Rapid Detection of Baijiu Alcohol Content Based on NIR and SNV-UVE-PLS. Proceedings of the 2023 CAA Symposium on Fault Detection, Supervision and Safety for Technical Processes (SAFEPROCESS).

[43] Luo Q., Tuo X.G., Zhang G.Y., Luo L., Zhai S., Zeng X.L. (2023). Application of Near-infrared Spectroscopy Combined with iPLS_SPA Band Screening in the Prediction Model of Yellow Water Alcohol Content. Mod. Food Sci. Technol..

[44] Chen L.Y., Zhao Z.G., Liu F. (2018). mRMR-based wavelength selection for quantitative detection of Chinese yellow wine using NIRS. Anal. Methods.

[45] Maskell P.D., Holmes C., Huismann M., Reidb S., Carrc M., Jonesa B.J., Maskell D.L. (2018). The influence of alcohol content variation in UK packaged beers on the uncertainty of calculations using the Widmark equation. Sci. Justice.

[46] Almeida V.E., Neto J.F.B., Bezerra T.K.A., Silva V.P., Veras G., Oliveira Ramos R., Sousa Fernandes D.D. (2025). Quantification of alcohol content and identification of fraud in traditional cachaças using NIR spectroscopy. Food Chem..

[47] Rimba C.I., Yumiko Y., Kayu O., Kazunori T. (2022). Measuring the Alcohol Content in Shochu Moromi by Near-infrared Spectroscopy. Bunseki Kagaku.

[48] Dos Santos C.A.T., Páscoa R.N.M.J., Porto P.A.L.S., Cerdeira A.L., González-Sáiz J.M., Pizarro C., Lopes J.A. (2018). Raman spectroscopy for wine analyses: A comparison with near and mid infrared spectroscopy. Talanta.

[49] Saha K.K., Al Riza D.F., Ogawa Y., Suzuki T., Sugimoto T., Kondo N. (2022). Assessment of chalkiness index of Sake rice using transmission imaging. Spectrochim. Acta Part A Mol. Biomol. Spectrosc..

[50] Lv Y., Wu H.S., Tang R., Zhao M.F., Li Y.F., Wei F.Y., Ge W.H., Li C.Y., Du W.F. (2023). Rapid quality identification of the whole wine-steamed process of Polygonati Rhizome by chromaticity and near-infrared spectroscopy. Infrared Phys. Technol..

[51] Shen F., Wu Q., Wei Y.Q., Liu X., Tang P.A. (2017). Evaluation of near-infrared and mid-infrared spectroscopy for the determination of routine parameters in Chinese rice wine. J. Food Process. Preserv..

[52] Kljusurić J.G., Boban A., Mucalo A., Budić-Leto I. (2022). Novel Application of NIR Spectroscopy for Non-Destructive Determination of ‘Maraština’ Wine Parameters. Foods.

[53] Zhu L.N., Wang Q. (2023). Rapid Analysis by NIR about Physicochemical Indexes of Fermented Grains of Nongxiang Baijiu in Northern China. Liquor. Mak..

[54] Huang Y.X., Tian J.P., Hu X.J., Yang H.L., Xie L.L., Zhou Y.F., Xia Y.Y., Huang D., He K.P. (2025). Predicting the composition of aroma components in Baijiu using hyperspectral imaging combined with a replication allocation strategy-enhanced stacked ensemble learning model. Spectrochim. Acta Part A Mol. Biomol. Spectrosc..

[55] Han S.H., Zhang W.W., Li X., Li P.Y., Liu J.X. (2016). Determination of three alcohols in Chinese Dukang Base liquor by FT-NIR spectroscopy. Food Anal. Methods.

[56] Su P.F., Liu L.L., Yan Z.K., Zhang P.F., Zhang W.G. (2022). Establishment of NIR Rapid Analysis Model of Ethyl Acetate Index in Feng Flavour Base Liquor. Liquor. Mak..

[57] Liao L., Zhang G.Y., Zou Y.F., Zhu X.M., Peng H.B., Zhang W., Li Y. (2025). Preliminary Exploration and Application Research on the Model of Gathering Distillate According to the Quality Based on Fourier Transform Near Infrared Spectroscopy. China Brew..

[58] Lambrecht K., Nieuwoudt H., Toit W., Aleixandre-Tudo J.L. (2022). Moving towards in-line monitoring of phenolic extraction during red wine fermentations using infra-red spectroscopy technology. Influence of sample preparation and instrumentation. J. Food Compos. Anal..

[59] Manzano J.I., Cozzolino D., Vilanova M. (2025). Optimisation of the optical path-length for the measurement of volatile compounds in wine using ultraviolet/visible/and near-infrared spectroscopy. Int. J. Food Sci. Technol..

[60] Pettinelli S., Alfieri G., Bianchi A., Baris F., Chinnici F., Mencarelli F., Mencarelli A., Modesti M. (2025). Fortified or passito sweet wines from Aleatico grapes subjected to different dehydration conditions: Chemical and aromatic profile using destructive and non-destructive analyses. OENO One.

[61] Bai X.B., Xu Y.Q., Chen X.L., Dai B.X., Tao Y.S., Xiong X.L. (2023). Analysis of Near-Infrared Spectral Properties and Quantitative Detection of Rose Oxide in Wine. Agronomy.

[62] Cayuela J.A., Puertas B., Cantos-Villar E. (2017). Assessing wine sensory attributes using Vis/NIR. Eur. Food Res. Technol..

[63] Santos C.D., Mesa N.F.O., Freire M.S., Ramosc R.P., Mederos B.J.T. (2019). Development of predictive models for quality and maturation stage attributes of wine grapes using vis-nir reflectance spectroscopy. Postharvest Biol. Technol..

[64] Fernández G.M.P., Simon E., Gibert A., Miranda J., Alcoba E.R., Martínez O., Cerezo E.V., Bustamante M.A. (2021). Gluten assessment in beers: Comparison by different commercial elisa kits and evaluation of nir analysis as a complementary technique. Foods.

[65] Huynh Q.T., Nguyen U.D., Nguyen H.N. Detect Level of Methanol in Alcohol Using Near-Infrared (NIR) Spectrometer Imaging. Proceedings of the 2023 International Conference on Advanced Technologies for Communications (ATC).

[66] Su Y.Y., Yang Y., Kilmartin P.A., Araujo L.D. (2025). Inter-regional characterization of New Zealand pinot noir wines: Assessing geographical origin through mid-FTIR and phenolic profile analysis. Food Res. Int..

[67] Machyňáková A., Schneider M.P., Khvalbota L., Vyviurska O., Špánik I., Gomes A.A. (2021). A fast and inexpensive approach to characterize Slovak Tokaj selection wines using infrared spectroscopy and chemometrics. Food Chem..

[68] Harris N., Gonzalez V.C., Barnes C., Fuentes S. (2022). Non-invasive digital technologies to assess wine quality traits and provenance through the bottle. Fermentation.

[69] Yu J., Zhan J.C., Huang W.D. (2017). Identification of wine according to grape variety using near-infrared spectroscopy based on radial basis function neural networks and least-squares support vector machines. Food Anal. Methods.

[70] Han S.H., Zhang W.W., Li X., Li P.Y., Liu J.X., Luo D.L., Xu B.C. (2016). Rapid determination of ethyl pentanoate in liquor using Fourier transform near-infrared spectroscopy coupled with chemometrics. Spectrosc. Lett..

[71] Zong X.Y., Peng H.B., Wu J.H., Sheng X.F., Li L. (2022). Study on the year and grade of Luzhou-flavor liquor by chemometrics and NIR. Packag. Food Mach..

[72] Gonzalez V.C., Fuentes S., Torrico D., Howell K., Dunshea F.R. (2018). Assessment of beer quality based on foamability and chemical composition using computer vision algorithms, near infrared spectroscopy and machine learning algorithms. J. Sci. Food Agric..

[73] Gonzalez V.C., Fuentes S. (2020). A digital approach to model quality and sensory traits of beers fermented under sonication based on chemical fingerprinting. Fermentation.

[74] Hu Q., Feng X.X., Wang L., Zhang L.L., Chen X.P., Wang Y.Y., Xie G.F., Peng Q. (2025). Rapid discrimination of Chinese rice Wine (Huangjiu) from various regions using Benchtop-NIR and Micro-NIR spectrometers in conjunction with chemometrics. Infrared Phys. Technol..

[75] Peng Q., Chen J.L., Meng K., Zheng H.J., Chen G.Q., Xu X., Lin Z.C., Xie G.F. (2022). Rapid detection of adulteration of glutinous rice as raw material of Shaoxing Huangjiu (Chinese Rice Wine) by near infrared spectroscopy combined with chemometrics. J. Food Compos. Anal..

[76] Jakubíková M., Sádecká J., Kleinová A. (2018). On the use of the fluorescence, ultraviolet–visible and near infrared spectroscopy with chemometrics for the discrimination between plum brandies of different varietal origins. Food Chem..

[77] Littarru E., Modesti M., Alfieri G., Pettinelli S., Floridia G., Bellincontro A., Sanmartinc C., Brizzolara S. (2025). Optimizing the winemaking process: NIR spectroscopy and e-nose analysis for the online monitoring of fermentation. J. Sci. Food Agric..

[78] Alfieri G., Riggi R., Modesti M., Bellincontro A., Renzi F., leixandre-Tudo J.L. (2025). Feasibility assessment of a low-cost near-infrared spectroscopy-based prototype for monitoring polyphenol extraction in fermenting musts. J. Sci. Food Agric..

[79] Vann L., Layfield J.B., Sheppard J.D. (2017). The application of near-infrared spectroscopy in beer fermentation for online monitoring of critical process parameters and their integration into a novel feedforward control strategy. J. Inst. Brew..

[80] Hua Y., Yuan L.M., Zhang H.N., Li L.M. (2020). Rapid Measurement of the Polyphenol Content in Fruit-Wine by Near Infrared Spectroscopy Combined with Consensus Modeling Approach. Spectrosc. Spectr. Anal..

[81] Wang B.Q., Peng B.Z. (2017). A feasibility study on monitoring residual sugar and alcohol strength in kiwi wine fermentation using a fiber-optic FT-NIR spectrometry and PLS regression. J. Food Sci..

[82] Kasemsumran S., Boondaeng A., Ngowsuwan K., Jungtheerapanich S., Apiwatanapiwat W., Janchai P., Vaithanomsat P. (2023). Mid-infrared and near-infrared spectroscopies to classify improper fermentation of pineapple wine. Chem. Pap..

[83] Sartor S., Toaldo I.M., Panceri C.P., Caliaric V., Lunae A.S., Goise J.S., Bordignon-Luiz M.T. (2019). Changes in organic acids, polyphenolic and elemental composition of rosé sparkling wines treated with mannoproteins during over-lees aging. Food Res. Int..

[84] Aleixandre T.J.L., Nieuwoudt H., Aleixandre J.L., du Toit W. (2018). Chemometric compositional analysis of phenolic compounds in fermenting samples and wines using different infrared spectroscopy techniques. Talanta.

[85] Hu X.Z., Liu S.Q., Li X.H., Wang C.X., Ni X.L., Liu X., Wang Y., Liu Y., Xu C.H. (2019). Geographical origin traceability of Cabernet Sauvignon wines based on Infrared fingerprint technology combined with chemometrics. Sci. Rep..

[86] Helfer G.A., Barbosa J.L.V., Hermes E., Fagundes B.J., Santos R.O., Costa A.B. (2022). The application of parallel processing in the selection of spectral variables in beer quality control. Food Chem..

[87] Molla N., Bakardzhiyski I., Manolova Y., Bambalov V., Cozzolino D., Antonov L. (2017). The effect of path length on the measurement accuracies of wine chemical parameters by UV, visible, and near-infrared spectroscopy. Food Anal. Methods.

[88] Chen L.Y., Zhao Z.G., Liu F. (2017). An updating method of NIR model based on characteristic wavelength for yellow rice wine detection. Spectrosc. Spectr. Anal..

[89] Debebe A., Temesgen S., Abshiro M.R., Chandravanshi B.S. (2017). Partial least squares− near infrared spectrometric determination of ethanol in distilled alcoholic beverages. Bull. Chem. Soc. Ethiop..

[90] Fulgêncio A.C.C., Resende G.A.P., Teixeira M.C.F., Botelho B.G., Sena M.M. (2022). Screening method for the rapid detection of diethylene glycol in beer based on chemometrics and portable near-infrared spectroscopy. Food Chem..

[91] França L., Grassi S., Pimentel M.F., Amigo J.M. (2021). A single model to monitor multistep craft beer manufacturing using near infrared spectroscopy and chemometrics. Food Bioprod. Process..

[92] Aleixandre-Tudo J.L., Nieuwoudt H., du Toit W. (2019). Towards on-line monitoring of phenolic content in red wine grapes: A feasibility study. Food Chem..

[93] Rouxinol M.I., Martins M.R., Murta G.C., Mota Barroso J., Rato A.E. (2022). Quality Assessment of Red Wine Grapes through NIR Spectroscopy. Agronomy.

[94] Liu J., Dong X., Han S., Xie A., Li X., Li P., Xu B., Luo D. (2021). Determination of ethyl octanoate in Chinese liquor using FT-NIR spectroscopy. Int. Food Res. J..

[95] Anjos O., Caldeira I., Roque R., Pedro S.I., Lourenço S., Canas S. (2020). Screening of Different ageing technologies of wine spirit by application of near-infrared (NIR) spectroscopy and volatile quantification. Processes.

[96] Tzanova M., Atanassova S., Atanasov V., Grozeva N. (2020). Content of polyphenolic compounds and antioxidant potential of some Bulgarian red grape varieties and red wines, determined by HPLC, UV, and NIR spectroscopy. Agriculture.

[97] Ripoll G., Vazquez M., Vilanova M. (2017). Ultraviolet–visible-near infrared spectroscopy for rapid determination of volatile compounds in white grapes during ripening. Ciênc. Téc. Vitiviníc..

[98] Hanousek-Čiča K., Pezer M., Mrvčić J., Stanzer D., Cacic J., Jurak V., Krajnovic M., Kljusuric J.G. (2019). Identification of phenolic and alcoholic compounds in wine spirits and their classification by use of multivariate analysis. J. Serbian Chem. Soc..

[99] Gonzalez V.C., Fuentes S. (2020). Beer aroma and quality traits assessment using artificial intelligence. Fermentation.

[100] Yan Y.J., Ren J.C., Tschannerl J., Zhao H.M., Harrison B., Jack F. (2021). Nondestructive phenolic compounds measurement and origin discrimination of peated barley malt using near-infrared hyperspectral imagery and machine learning. IEEE Trans. Instrum. Meas..

[101] Hu L.Q., Yin C.L., Ma S., Liu Z.M. (2018). Rapid detection of three quality parameters and classification of wine based on Vis-NIR spectroscopy with wavelength selection by ACO and CARS algorithms. Spectrochim. Acta Part A Mol. Biomol. Spectrosc..

[102] Fuentes S., Torrico D.D., Tongson E., Viejo C.J. (2020). Machine learning modeling of wine sensory profiles and color of vertical vintages of pinot noir based on chemical fingerprinting, weather and management data. Sensors.

[103] Kalinowska K., Tobiszewski M. (2023). Green, simple analytical method for total biogenic amines content determination in wine using spectrophotometry. Food Chem..

